# Blood-letting therapy for the common cold: A protocol for a systematic review of controlled trials

**DOI:** 10.1097/MD.0000000000009315

**Published:** 2017-12-22

**Authors:** Ju Ah Lee, Minna Hong, Myeong Soo Lee, Seong Hoon Yoon, Jun-Yong Choi

**Affiliations:** a Department of Korean Internal Medicine, College of Korean Medicine, Gachon University; b The Graduate School of Pusan National University, Busan; c Department of Internal Medicine, Korean Medicine Hospital of Pusan National University, Yangsan; d Clinical Research Division, Korea Institute of Oriental Medicine, Daejeon; e Lung Cancer Clinic, Pulmonary Medicine Center, Pusan National University Yangsan Hospital; f School of Korean Medicine, Pusan National University, Yangsan, Republic of South Korea.

**Keywords:** blood-letting, common cold, protocol, systematic review

## Abstract

**Background:**

Many people experience the common cold, but there is currently no special treatment. For this reason, complementary and alternative medicine (CAM) therapies are used to improve the symptoms of the common cold. Blood-letting therapy (BL) is a CAM therapy that has been used for over 2000 years to treat various diseases. However, few studies have provided evidence for the efficacy and safety of BL for the common cold. This study aims to assess the effectiveness and safety of BL for the common cold.

**Methods and analysis:**

A total of 11 databases will be searched for studies conducted through June 2017. We will include randomized controlled trials assessing BL for the common cold. All randomized controlled trials on BL or related interventions will be included. Risk of bias will be assessed using the Cochrane Risk of Bias Assessment Tool, while confidence in the accumulated evidence will be evaluated using the Grading of Recommendations Assessment, Development and Evaluation instrument.

**Ethics and dissemination:**

This systematic review will be published in a peer-reviewed journal and will also be disseminated electronically and in print. The review will be updated to inform and guide healthcare practices.

## 1 Introduction

The common cold is an upper respiratory tract infection that can affect any part of the respiratory mucosa.^[1]^ Symptoms of the common cold include rhinorrhea, nasal congestion, fever, headache, and muscle pain.^[2]^ In addition, half of the patients experience sore throat, and 40% experience cough.^[1]^ The symptoms peak within 2 to 3 days after infection and persist for average of 7 to 10 days, but depending on the proportion of patients, some symptoms may be present for 3 weeks.^[3,4]^

The common cold is self-limited; therefore, treatment focuses on symptom relief. Various medications have been used to treat symptoms of the common cold,^[2]^ but there are few effective treatments that reduce the symptoms. Nonsteroidal antiinflammatory drugs (NSAIDs) can reduce pain symptoms^[2]^ and improve symptoms in adults^[5]^ but do not reduce the overall duration.^[2]^ The antiviral drug oseltamivir barely shortens the duration of cold symptoms in healthy adults,^[5]^ and antibiotics are not effective in either children or adults.^[2,5]^ For these reasons, complementary and alternative medicine (CAM) therapies are used by many patients to improve the symptoms of the common cold.^[5]^

Cupping therapy is a CAM therapy that has been used for at least 2000 years.^[6]^ It has been practiced in China, the Middle East, and Europe.^[7]^ In particular, blood-letting therapy (BL), which is also called blood-letting cupping or wet cupping, is the most preferred cupping method.^[8]^ It can be used to treat sudden hypertension and to discharge pus caused by the heat and stagnation of blood.^[8]^ At present, many clinical trials have evaluated the effectiveness of BL on a variety of diseases including herpes zoster, facial paralysis, and acne.^[6]^ However, no useful studies have been found to summarize the evidence and evaluate the efficacy and safety of BL for the common cold. Therefore, the purpose of this study is to systematically review the literature to evaluate the efficacy and safety of BL for common cold. This review will also provide various options for common cold treatment.

## 2 Methods

### 2.1 Study registration

This study will follow the guidelines outlined in the Preferred Reporting Items for Systematic Reviews and Meta-Analysis statement for meta-analyses of healthcare interventions. The protocol for this systematic review has been registered on PROSPERO 2015 under the number CRD42017069505.

### 2.2 Data sources

The following databases will be searched from inception to the present date: MEDLINE, EMBASE, the Cochrane Central Register of Controlled Trials, AMED, and CINAHL. We will also search 6 Korean medical databases (ie, OASIS, the Korean Traditional Knowledge Portal, the Korean Studies Information Service System, KoreaMed, and the Korean Medical Database and DBPIA) and 3 Chinese databases, including CNKI (ie, the China Academic Journal, the China Doctoral Dissertations and Master's Theses Full-text Database, and the China Proceedings of Conference Full-Text Database and the Century Journal Project), Wanfang, and VIP. In addition, we will search a Japanese database and conduct nonelectronic searches of conference proceedings, as well as our own article files.

### 2.3 Types of studies

Studies of prospective randomized controlled trials that include BL as the sole treatment or as an adjunct to other treatments and that provide the same treatment to the control and intervention groups will be included. Trials comparing BL with any type of control intervention will also be included. No language restrictions will be imposed.

### 2.4 Types of participants

The participants will include any individuals who want to receive treatment for the common cold, regardless of age, gender, and race.

### 2.5 Types of interventions

Studies that evaluate any type of invasive BL will be included. Control interventions may include treatments such as general conventional care (including pharmacological interventions such as ant infective or antipyretic drugs), BL, or waiting list care. Accordingly, we will include all pragmatic trials that compare BL with any other treatment (eg, drugs). We will exclude randomized controlled trials that compare one form of BL with a different form of BL. We will also exclude trials that compare BL plus another active treatment with the same active treatment alone.

### 2.6 Data extraction and quality assessment

Two authors (JAL and MH) will perform the data extraction and quality assessment using a predefined data extraction form. Risk of bias will be assessed using the Cochrane Handbook Risk of Bias Assessment Tool version 5.1.0, which considers random sequence generation, allocation concealment, blinding of participants and personnel, blinding of outcome assessment, incomplete outcome data, selective reporting, and other sources of bias.^[9]^ The results of the assessments will be presented using scores of “L,” “U,” and “H,” with “L” indicating a low risk of bias, “U,” an uncertain risk of bias, and “H,” a high risk of bias. Disagreements will be resolved by discussion among all authors. When disagreements regarding selection cannot be resolved through discussion, an arbiter (JYC) will make the final decision.

## 3 Data collection and synthesis

### 3.1 Outcome measures

#### 3.1.1 Primary outcomes

The primary outcomes will be the therapeutic effects of treatment on the common cold.

#### 3.1.2 Secondary outcomes

The secondary outcomes will include safety based on adverse effects. In addition, the improvement of symptoms (eg, high fever and pain) will be included as secondary outcomes.

### 3.2 Assessment of bias in the included studies

We will independently assess the bias of the included studies according to the criteria in the Cochrane Handbook, version 5.1.0; these criteria include random sequence generation, allocation concealment, blinding of participants and personnel, blinding of outcome assessment, incomplete outcome data, selective reporting, and other sources of bias.^[9]^

### 3.3 Data synthesis

Differences between the intervention and control groups will be assessed. Mean differences (MDs) with 95% confidence intervals (CIs) will be used to measure the effects of treatment for continuous data. We will convert other forms of data into MDs. For outcome variables on different scales, we will use standard MDs (SMDs) with 95% CIs. For dichotomous data, we will present treatment effects as relative risks (RRs) with 95% CIs; other binary data will be converted into RR values.

All statistical analyses will be conducted using Cochrane Collaboration's software program Review Manager version 5.3. (Copenhagen, The Nordic Cochrane Centre, the Cochrane Collaboration, 2012) for Windows. We will contact the corresponding authors of studies with missing information to acquire and verify the data whenever possible. When appropriate, we will pool the data across studies to conduct a meta-analysis using fixed- or random-effect models. We will use GRADEpro software from Cochrane Systematic Reviews to create a summary of findings table.

### 3.4 Unit of analysis issues

For crossover trials, data from the first treatment period will be used. For trials that assessed more than one control group, the primary analysis will combine data from each control group. Subgroup analyses of the control groups will be performed. Each patient will be counted only once in the analyses.

### 3.5 Addressing missing data

Intention-to-treat analyses including all randomized patients will be performed. For patients with missing outcome data, last observation carry-forward analysis will be performed. When individual patient data are initially unavailable, we will review the original source or the published trial reports for these data.

### 3.6 Assessment of heterogeneity

Based on the data analysis, we will use random- or fixed-effect models to conduct the meta-analysis. Chi-squared and I-squared tests will be used to evaluate the heterogeneity of the included studies. *I*^2^ values >50 will indicate high heterogeneity. When heterogeneity is observed, subgroup analyses will be conducted to explore the possible causes.^[10]^

### 3.7 Assessment of reporting biases

Funnel plots will be generated to detect reporting biases when a sufficient number of included studies (at least 10 trials) is available.^[11]^ However, as funnel plot asymmetries are not equivalent to publication biases, we will aim to determine the possible reasons for any asymmetries in the included studies, such as small study effects, poor methodological quality, and true heterogeneity.^[11,12]^

## 4 Discussion

BL is a CAM therapy commonly used in the Middle East, Asia, and Europe and is the 3rd most common treatment in Saudi Arabia.^[13]^ Some studies have suggested that BL is an effective treatment for musculoskeletal pain, hypertension, chronic obstructive pulmonary disease (COPD), and Behcet disease.^[14]^ In addition, one case series study has been reported indicating that fire insertion cupping therapy has an antipyretic effect in the treatment of high fever caused by upper respiratory tract infection.^[15]^ However, no systematic review has been conducted that provides evidence of the effectiveness and safety of BL for the common cold. We have presented a protocol for a systematic review of BL for the common cold. We hope that this study will form the basis to conduct additional research and provide evidence for the use of BL for the common cold.
